# Cytochrome P450 2A6 whole-gene deletion (CYP2A6*4) polymorphism reduces risk of lung cancer: A meta-analysis

**DOI:** 10.18332/tid/122465

**Published:** 2020-06-02

**Authors:** Fadzrul H. Johani, Mohd S. A. Majid, Muhammad H. Azme, Azmawati M. Nawi

**Affiliations:** 1 Department of Community Health, Faculty of Medicine, University Kebangsaan Malaysia, Cheras, Malaysia

**Keywords:** CYP2A6*4, Cytochrome P450 2A6, lung cancer, meta-analysis, polymorphism

## Abstract

**INTRODUCTION:**

Lung cancer is the most commonly diagnosed cancer worldwide and is the leading cause of cancer death. Smoking is a major contributor to the pathogenesis of lung cancer. Cytochrome P450 2A6 (*CYP2A6*) is responsible for the metabolic activation of most tobacco carcinogens. *CYP2A6* genetic polymorphism can cause variations in the human metabolism of xenobiotics. We performed this meta-analysis to determine the association between whole-gene *CYP2A6* deletion polymorphism (CYP2A6*4) and lung cancer risk.

**METHODS:**

The PubMed, SAGE, Science Direct, the Cochrane Library and Ovid databases were searched for observational studies before October 2018. Methodological quality was assessed using the Newcastle-Ottawa Quality Assessment Scale (NOS).

**RESULTS:**

Nine case-control studies involving 4385 lung cancer cases and 4142 controls were included in the analysis. The random-effects model was used to combine results from individual studies. The pooled odds ratio was 0.39 (95% CI: 0.27–0.56). There was no heterogeneity across studies (χ^2^=2.49, p=0.96, I^2^=0%).

**CONCLUSIONS:**

Current evidence from the case-control studies suggests that the *CYP2A6* whole-gene deletion polymorphism decreases the risk of lung cancer. Further research is needed to identify any potential confounding factors that may impact this association.

## INTRODUCTION

In 2018, there were an estimated 18.1 million new cancer cases and 9.6 million cancer deaths, worldwide. Lung cancer is the most commonly diagnosed cancer worldwide, constituting 11.6% of total cancer cases, and is the leading cause of cancer death (18.4% of total cancer deaths)^1^.

Smoking is a major contributor to the pathogenesis of lung cancer^2^. Even exposure to secondhand tobacco smoke increases the risk of developing lung cancer among non-smokers^3,4^. The pro-carcinogens specific to tobacco are clearly implicated in the progression of lung malignancies^5,6^. However, not all smokers will eventually develop lung cancer in their lifetime. These individual differences in lung cancer morbidity could be due to other determinants such as genetic susceptibility^7,8^ and environmental factors^9-11^. Thus, it is important to identify the genetic variants that influence the risk of lung cancer initiation^12,13^.

Most tobacco carcinogens require metabolic activation, which is mainly executed by the cytochrome P450 (CYP) enzymes^14^. Electrophile agents with short lifespans (which are produced in metabolic activation) cross-react with DNA, causing DNA damage and initiating tumours^15,16^. Among the CYP isozymes, CYP family 2 subfamily A member 6 (*CYP2A6*) is responsible for nicotine metabolism and for the metabolic activation of the tobacco-specific pro-carcinogens *N*-nitrosamine *N*-nitrosonornicotine (NNN) and 4-(methylnitrosoamino)-1-(3-pyridyl)-1-butanone (NNK), which can eventually contribute to the progression of lung cancer^5^.

Genetic polymorphism is defined as the inheritance of a trait controlled by a single genetic locus with two alleles in which the least common allele has a frequency of approximately ≥1% that can cause variation in the DNA sequence in individuals, groups, or populations^17^. The difference in DNA sequence may not alter the overall product sufficiently enough to produce a different protein but may affect the specific activity of the enzyme, and binding efficiencies such as those for transcription factors or membrane proteins, or other features and function^18^. Thus, Cytochrome P450 2A6 (*CYP2A6*) polymorphism can influence how humans metabolize xenobiotics.

Over the past two decades, several studies have assessed the association between *CYP2A6* polymorphism, including whole-gene deletion of *CYP2A6* on allele 4 (CYP2A6*4), and the risk of lung cancer among different ethnic populations, but the results have been inconsistent^12,19,20^. Therefore, the present meta-analysis was performed to determine the association between *CYP2A6* whole-gene deletion (CYP2A6*4) polymorphism and lung cancer risk.

## METHODS

### Literature search

This meta-analysis was performed based on the PRISMA (Preferred Reporting Items for Systematic Reviews and Meta-Analyses) statement^21^. A literature search was performed on five databases, namely PubMed, SAGE, ScienceDirect, the Cochrane Library, and Ovid. The search strategy utilized the PICO (Population, Intervention, Comparison, Outcome) framework to improve searching for clinical questions^22^. The search terms used were: [‘cytochrome P450 2A6’ OR ‘*CYP2A6*’] AND [‘lung cancer’ OR ‘pulmonary cancer’ OR ‘respiratory cancer’]. The search was not restricted to any duration or timeline.

### Study selection

Two pairs of reviewers conducted the study selection in two phases after duplicate studies had been excluded. In the first phase, two reviewers (MSAM and MHA) independently screened the titles and abstracts of potential articles to be included in the study. During this phase, irrelevant studies were excluded, and a third reviewer (FHJ) resolved any disagreements. In the second phase, full-text articles were retrieved for detailed evaluation. Studies were included if they were published prior to October 2018 and met the following criteria: 1) observational study design, 2) presence of *CYP2A6* whole-gene deletion (CYP2A6*4) polymorphism, and 3) presence of lung cancer. We excluded studies that were not published in English, reviews, case reports or animal studies, or if the full text was not available.

### Data extraction

Articles that met the inclusion and exclusion criteria were retained for a full review. The characteristics of each study were examined and included study design, study location, type of population, sample size, sex, smoking status, matching criteria, genotyping method and genotype, and risk estimate value.

### Assessment of methodological quality

Two authors assessed the quality of the selected articles independently, using the Newcastle-Ottawa Quality Assessment Scale (NOS)^23^ to examine for the concordance and average NOS score for each study. The NOS is widely used for quality assessment of observational studies^24,25^. It evaluates three components to quantify study quality, i.e. selection of study subjects, comparability of study groups and exposure or outcome ascertainment, which consists of eight items with a maximum score of 9 for each study. The scores of each item indicate the methodological quality of the study. A study is categorized as being of high, moderate or low quality, based on a total score of 7–9, 4–6 and 0–3, respectively^26^.

### Meta-analysis

The random-effects model^27^ was used to estimate the pooled effect size from the included studies. Odds ratio (OR) with 95% confidence interval (CI) and a statistical measure of heterogeneity (χ^2^ and I^2^) were calculated using Review Manager 5.3^28^. All selected studies were included in the meta-analysis. Subgroup and sensitivity analyses were performed if p<0.10 and I^2^ ≥50%.

## RESULTS

**Figure 1:**
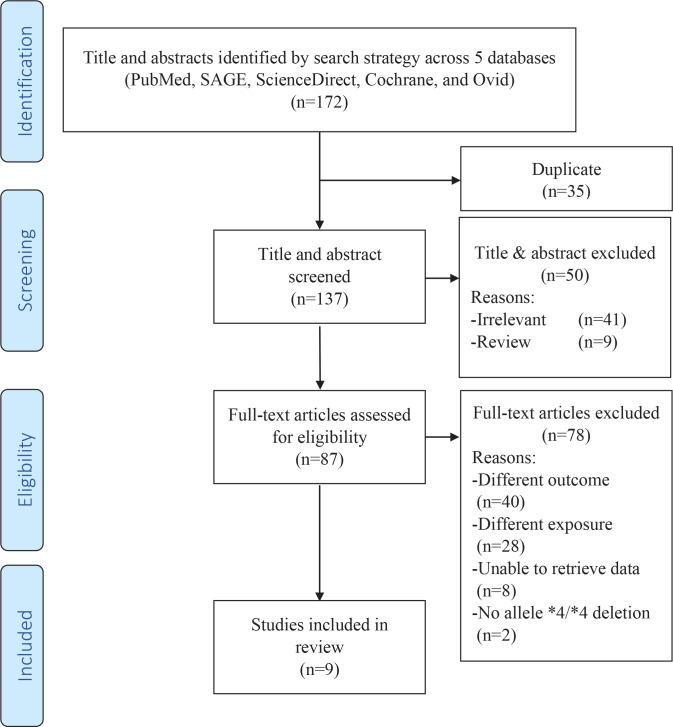
Flow chart of studies selection

The search strategy returned a total of 172 articles. Initial screening excluded 35 articles due to duplication. Further screening of titles and abstracts excluded 41 irrelevant studies and nine review articles. The eligibility of the remaining 87 articles was assessed: 40 were excluded for studying different outcomes such as smoking behaviour, 28 were excluded for studying different exposures such as other CYP450 genotypes, eight were excluded as we were unable to extract raw data related to the whole deletion of allele *4/*4, while two articles were excluded because we did not find data related to the whole deletion of allele *4/*4 in the studies. The remaining nine articles^12,20,29-35^ were included in the meta-analysis. Figure 1 depicts the flow diagram describing article retrieval based on the PRISMA flow diagram^21^. The studies were carried out in Japan, China, Bangladesh, and Italy. Eight studies used Asian samples and only one study^34^ used Caucasian samples. Tables 1 and 2 present the characteristics of the included studies.

**Table 1:** Characteristics of studies included in the meta-analysis

*Study*	*Year Country & Population*	*Study design (cases/controls)*	*Gender, Smoking status*	*Matching criteria*	*Genotyping methods*	*Histological type (%)*	*CYP2A6 genotype*	*Crude OR (95% CI)*
Hosono et al.^12^	2015 Japan Asian	Case-control 110/132	Both All smoker	Age, sex	PCR-Goodz and Tyndale	SQCC (100)	Group 1: 1/1 Group 2: 1/7, 1/9, 1/10, 1/13, 1/15, 8/9 Group 3: 1/4, 1/41, 1/567C>T, 7/9, 7/11, 7/13, 9/9, 9/11 Group 4: 4/7, 4/9, 4/13, 4/18, 7/567C>T Group 5: 4/4, 4/5	Group 1: 1 (Ref.) Group 2: 1.00 (0.49–2.07) Group 3: 0.71 (0.35–1.45) Group 4: 0.13 (0.04–0.45) Group 5: 0.15 (0.03–0.82)
Islam et al.^29^	2013 Bangladesh Asian	Case-control 106/116	Both Mixed	Age, sex, smoking status	PCR-RFLP	SQCC (43.39) AC (34.91 SCC (18.87) ASQC (0.94)	Group 1: 1A/1A, 1A/1B1, 1B1/1B1. Group 2: 1A/4, 1B1/4. 4/4	Group 1: 1 (Ref.) Group 2: 0.40 (0.17–0.91)
Tamaki et al.^30^	2011 Japan Asian	Case-control 192/203	Both Mixed	Age, sex	PCR- Oscarson	AC (41.7) SQCC (24.5) SCC (21.4) ASQC (2.1) LCC (0.5) Unknown (3.6)	Group 1: non-4/ non-4 Group 2: non-4/4 Group 3: 4/4	Group 1: 1 (Ref.) Group 2: 0.92 (0.60–1.42) Group 3: 0.36 (0.14–0.88)
Rotunno et al.^34^	2009 Italy Caucasian	Case-control 1859/2019	Both Mixed	Age, sex, area of residence	SNP Assays	AC (41) SQCC (25.6) SCC (10.2) Other (21.5) Unknown (1.8)	Group 1: T/T Group 2: T/A Group 3: A/A	Group 1: 1 (Ref.) Group 2: 0.74 (0.55–1.00) Group 3: 0.26 (0.04–1.94)
Fujieda et al.^31^	2004 Japan Asian	Case-control 1094/611	Both All smokers	No matching	PCR-RFLP	SQCC (26.9) SCC (12.2) AC (50.9) Unknown (10.0)	Group 1: 1/1 Group 2: 1/4, 1/7, 1/9, 1/10, 1/11 Group 3: 4/7, 4/9, 4/10, 4/11, 7/7, 7/9, 7/10, 9/9, 9/10, 9/11, 10/10 Group 4: 4/4	Group 1: 1 (Ref.) Group 2: 0.59 (0.44–0.79)^a^ Group 3: 0.52 (0.37–0.72)^a^ Group 4: 0.30 (0.16–0.57)^a^
Ariyoshi et al.^32^	2002 Japan Asian	Case-control 370/380	Both All smokers	No matching	PCR-Bell	SCC (11.9) SQCC (28.4) AC (52.1) Others (7.6)	Group 1: 1A/1A Group 2: 1A/1B Group 3: 1B/1B Group 4: 1A/4 Group 5: 1B/4 Group 6: 4/4	Group 1: 1 (Ref.) Group 2: 0.70 (0.46–1.07) Group 3: 0.60 (0.37–0.99) Group 4: 0.57 (0.33–0.97) Group 5: 0.56 (0.34–0.92) Group 6: 0.18 (0.06–0.50)
Tan et al.^20^	2001 China Asian	Case-control 151/326	Both Mixed	Age, sex	PCR-Oscarson	SCC (58.3) AC (31.1) Other (10.6)	Group 1: 1/1 Group 2: 1/4, 4/4	Group 1: 1 (Ref.) Group 2: 2.0 (1.2–3.2)
Miyamoto et al.^33^	1999 Japan Asian	Case-control 246/201	Both NA	No matching	NA	NA	Group 1: Wild/Wild Group 2: Wild/Conv. Group 3: Conv./Conv. Group 4: Wild/Del. Group 5: Conv./Del. Group 6: Del./Del.	Group 1: 1 (Ref.) Group 2: 0.59 (0.34–1.02) Group 3: 0.57 (0.30–1.08) Group 4: 0.29 (0.14–0.59) Group 5: 0.46 (0.23–0.92) Group 6: 0.25 (0.08–0.83)
Kamataki et al.^35^	1999 Japan Asian	Case-control 257/154	Both NA	No matching	PCR-RFLP	NA	NA	NA

a Adjusted for age and smoking habit.

PCR-RFLP: Polymerase Chain Reaction - Restriction Fragment Length Polymorphism. Del: Deletion-type, Conv: Conversion-type. SQCC: Squamous Cell Carcinoma. AC: Adenocarcinoma. SCC: Small Cell Carcinoma. ASQC: Adenosquamous Cell Carcinoma. LCC: Large Cell Carcinoma.

**Table 2:** Genotype frequencies of CYP2A6*4 in studies included in the meta-analysis

*Author*	*Year*	*Case Genotype*			*Control Genotype*		

		*4/*4	*4/non-*4	non-*4/non-*4	*4/*4	*4/non-*4	non-*4/non-*4
Hosono et al.^12^	2015	2	32	76	8	48	76
Islam et al.^29^	2013	1	8	97	4	18	94
Tamaki et al.^30^	2011	7	63	122	19	66	118
Rotunno et al.^34^	2009	2	101	1756	4	160	1855
Fujieda et al.^31^	2004	25	301	768	28	186	397
Ariyoshi et al.^32^	2002	5	98	267	19	117	244
Tan et al.^20^	2001	1	38	112	5	46	275
Miyamoto et al.^33^	1999	5	48	193	9	60	132
Kamataki et al.^35^	1999	2	-	255	6	-	148

**Figure 2:**
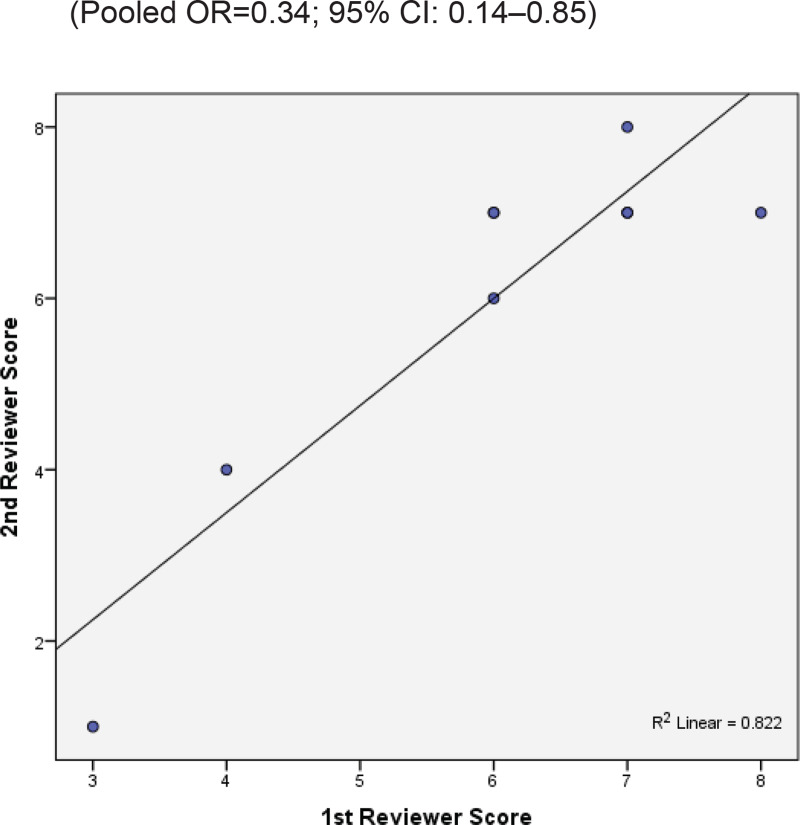
The research quality assessment of each study was assessed using NOS with two independent reviewers.

**Figure 3:**
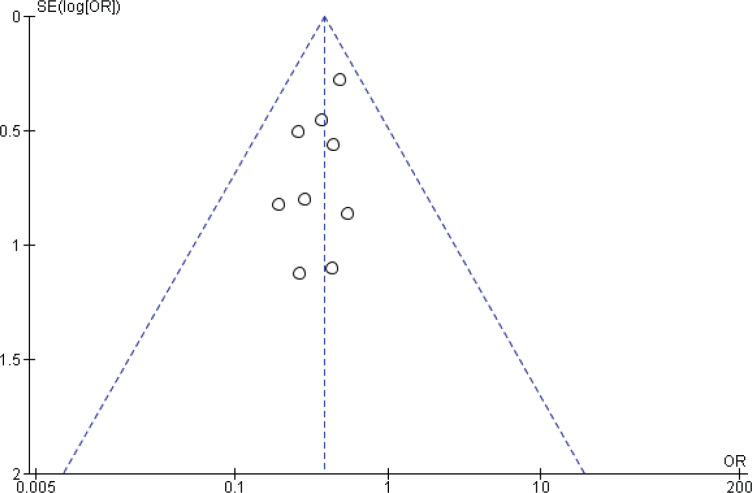
Funnel plot of the nine studies included in the meta-analysis

Two reviewers were independently assigned to score the NOS for each study individually. The correlation coefficient scores between the two authors were strong, with r = 0.91 (Figure 2). Overall, the studies had moderate methodological quality as scored on NOS (mean score: 6.0; range: 2.0–7.5). Four studies were of high quality (NOS score: 7.0– 9.0)^20,29,32,34^, four were of moderate quality (NOS score: 4.0–6.9)^21,31,32,34^, and only one study was of low quality (NOS score: <4.0)^35^. The shape and symmetry of the funnel plot of log OR from the nine studies indicated that there was no publication bias (Figure 3). All studies had a high precision value.

**Figure 4:**
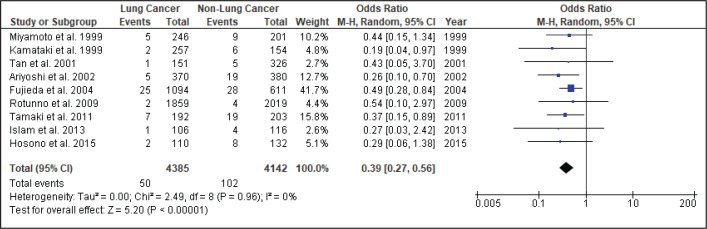
Forest plot analysis

**Figure 5:**
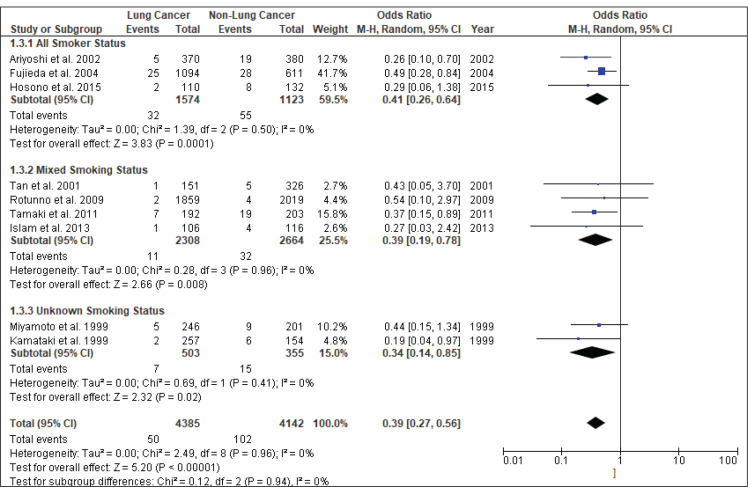
Subgroup analysis according to smoking status

Figure 4 shows the result of the meta-analysis using the random-effects model 28, which combined the nine studies to explore the association between the CYP2A6*4 polymorphism and the risk of lung cancer. The forest plot illustrates the spread of the studies’ risk estimates and their CIs in relation to the pooled OR of meta-analysis. The pooled OR estimates showed that the CYP2A6*4 whole-gene deletion polymorphism significantly reduced the risk of lung cancer (pooled OR=0.39; 95% CI: 0.27–0.56). No heterogeneity was found across the studies (pheterogeneity = 0.96, I2=0%). Subgroup analysis according to smoking status (Figure 5) showed that the pooled OR estimate of the CYP2A6*4 whole-gene deletion polymorphism remained significantly protective against lung cancer among ‘All Smoker Status’ (pooled OR=0.41; 95% CI: 0.26–0.64), ‘Mixed Smoking Status’ (pooled OR=0.39; 95% CI: 0.19–0.78) and ‘Unknown Smoking Status’.

## DISCUSSION

A comprehensive search of different databases was used to yield the most relevant results and incorporated the available epidemiologic evidence to explore the relationship between the CYP2A6*4 whole-gene deletion polymorphism and the risk of lung cancer. Nine case-control studies fulfilled the criteria addressing this issue. The analysis, involving 4385 lung cancer cases and 4142 controls, suggested that *CYP2A6* polymorphism significantly reduces the risk of lung cancer (pooled OR=0.39; 95% CI: 0.27–0.56), with homogeneity observed across studies (χ^2^=2.49, p=0.96, I^2^=0%). Thus, subgroup and sensitivity analyses were not explored.

There are two possible explanations for the finding of relative risk reduction as revealed by the analysis. First, *CYP2A6* is mainly found in the liver and other tissues such as the nasal epithelium, trachea, lung, and oesophagus^36,37^. *CYP2A6* metabolizes a few but specific xenobiotics that include nicotine and some tobacco specific nitrosamines that enter the human body. Metabolic activation by *CYP2A6* enzymes generally produces a short-lived electrophile agent that reacts with DNA, causing DNA damage and inducing a tumour^16^. In tobacco smoke, *CYP2A6* is the main enzyme activating the tobacco-specific *N*-nitrosamines NNK and NNN, which are pro-carcinogens^5,6^. Thus, in individuals with the inactive *CYP2A6* genotype, the *CYP2A6* enzyme might not affect metabolic activation of N-nitrosamines and subsequently reduce the risk of lung cancer. Second, *CYP2A6* is a major enzyme responsible for nicotine metabolism^38^, where inactive *CYP2A6* causes lower nicotine dependence and thus affects smoking behaviour^39-41^.

Of the included studies, only three studies^12,31,32^ recruited only smokers as participants; the remaining studies had mixed populations of smokers and never-smokers. The mixed-smoker status studies yielded non-significant findings or smaller risk estimates, which may be explained by the fact that the probability of lung cancer occurrence may be similar among never-smokers with different *CYP2A6* genotypes, where the resulting phenotype is not expressed in people who do not smoke. Thus, when the original studies included both smokers and non-smokers, the association between the *CYP2A6* polymorphism genotype and risk of lung cancer may have been attenuated. This may explain why some of the studies did not find any significant association^19,20,29,42^.

Besides that, cigarette smoking is strongly associated with squamous cell carcinoma compared to adenocarcinoma^43-45^. However, in the present meta-analysis, all but one study included different lung cancer histology types; Hosono et al.^12^ only recruited squamous cell carcinoma cases. The study by Islam et al.^29^ had the highest proportion of squamous cell carcinoma cases (43.39%), while the other studies had 24.5–28.4% squamous cell carcinoma cases^30-32,34^. These differences in the proportion of histological types might explain the discrepancy of the findings among the studies on *CYP2A6* polymorphism and lung cancer risk.

### Limitations

The present meta-analysis findings should be interpreted with caution. Six studies did not stratify the smoking status to assess the association between *CYP2A6* polymorphism and lung cancer. Therefore, the true relationship between *CYP2A6* polymorphism and risk of lung cancer in current-smokers and never-smokers could not be tested in these six studies. In addition, lung cancer might arise due to occupational carcinogen exposure, such as organic dust and silica dust^46,47^, which may confound the association between *CYP2A6* polymorphism and lung cancer risk. However, none of the included studies adjusted for occupational carcinogen hazard exposure originally. The relationship may be also affected by differences in the demographic characteristics and socioeconomic class of the respondents^48^, which some of the studies did not mention.

## CONCLUSIONS

The evidence from the case-control studies included in the present meta-analysis shows that people with CYP2A6*4 whole-gene deletion have a decreased risk of lung cancer. Further research is needed to identify any potential confounding factors that may impact this association.
